# Anticoagulant Therapy for Cancer-Associated Venous Thromboembolism after Cancer Remission

**DOI:** 10.3400/avd.oa.21-00022

**Published:** 2021-06-25

**Authors:** Nobuhiro Hara, Tetsumin Lee, Kentaro Mitsui, Masashi Nagase, Shinichiro Okata, Giich Nitta, Masakazu Kaneko, Yasutoshi Nagata, Toshihiro Nozato, Takashi Ashikaga

**Affiliations:** 1 Department of Cardiology, Musashino Red Cross Hospital, Musashino, Tokyo, Japan

**Keywords:** anticoagulation, therapy, cancer-associated VTE, cancer remission

## Abstract

**Objectives:** To examine the outcomes of anticoagulant therapy for patients with venous thromboembolism (VTE) with active cancer and the outcomes after cancer remission with and without anticoagulant therapy.

**Materials and Methods:** Of the 338 patients with cancer-associated VTE who received anticoagulant therapy, we evaluated therapeutic outcomes over 1 year for 112 patients whose cancers were in remission (cancer remission group) and 226 patients who continued cancer treatment (continued cancer treatment group). Further, the cancer remission group was divided into 89 and 23 patients who completed (completion of anticoagulation group) and continued (continued anticoagulation group) anticoagulant therapy, respectively. Treatment outcomes after completing anticoagulant therapy were compared between these two groups. The follow-up period was 1 year, and the endpoints were all-cause death, VTE recurrence, and bleeding events.

**Results:** The event-free survival rates were 99.1% and 42.9% in the cancer remission and continued cancer treatment groups, respectively. For treatment outcomes after the completion of anticoagulant therapy, the event-free survival rates were 98.9% and 87% in the completion of anticoagulation and continued anticoagulation groups, respectively (log rank, P=0.005).

**Conclusion:** When cancer is in remission, recurrence is low even if anticoagulant therapy is terminated after a certain period.

## Introduction

Patients with cancer are at high risk of developing venous thromboembolism (VTE). In Japan, 27% of patients with VTE of identifiable cause had malignancies.^1)^ However, there remains no clear standard duration of anticoagulant therapy for patients with cancer with VTE. Approximately 10% of patients experienced VTE recurrence within 1 year after discontinuation of anticoagulants and 50% within 10 years.^2,3)^ The Japanese Circulation Society (JCS) guidelines also recommend a longer period of therapy. On the other hand, the guidelines of the JCS, European Society of Cardiology,^4)^ and American Society of Clinical Oncology^5)^ recommend discontinuing anticoagulant therapy after cancer is in remission; however, there are few evidences on this. Thus, the present study aims to examine the clinical outcomes of anticoagulant therapy for patients with VTE with active cancer and the outcomes after cancer remission with and without anticoagulant therapy in a real-world setting.

## Materials and Methods

### Patient population

We reviewed medical records of patients at the Musashino Red Cross Hospital.

VTE was diagnosed when a thrombus was found on contrast-enhanced computed tomography (CT) or venous ultrasonography of the lower extremities. Patients with cancer-associated VTE included those receiving treatment for cancer, such as radiotherapy or chemotherapy; those scheduled to undergo cancer surgery; those with metastasis to other organs; and those with terminal cancer (expected life expectancy of ≤6 months) at the time of VTE diagnosis.^6)^ Cancer remission was defined as a state in which there was no cancer in the body at 1 year after the start of therapy for cancer-associated VTE and when at least 6 months had passed since the completion of surgery, chemotherapy, radiation therapy, or other treatments. Continued cancer treatment was defined as a state receiving treatment for cancer, such as radiotherapy or chemotherapy; those scheduled to undergo cancer surgery; those with metastasis to other organs; and those with terminal cancer (expected life expectancy of ≤6 months) at the time of 1 year after the start of therapy for cancer-associated VTE. The creatinine clearance was calculated using the Cockcroft–Gault formula. Heart disease was defined as an ischemic heart disease, arrhythmia, or heart failure history. Brain disease was defined as a history of cerebral infarction cerebral hemorrhage or epilepsy.

### Follow-up

The overall 338 patients with cancer-associated VTE who underwent anticoagulant therapy were divided into two groups: 112 patients with cancer remission after treatment (cancer remission group) and 226 patients who continued cancer treatment (continued cancer treatment group). In addition, the cancer remission group of 112 patients was divided into 89 and 23 patients who discontinued (completion of anticoagulation group) and continued (continued anticoagulation group) anticoagulant therapy, respectively. Clinical outcomes were examined in the completion of anticoagulation group at the 1-year follow-up period after the end of anticoagulant therapy. As a median duration of anticoagulant therapy in the completion of the anticoagulation group was 190 days, the continued anticoagulation group was examined 1 year after the 190th day from the start of oral administration. Anticoagulant therapy discontinuation was left to the discretion of the doctor.

The endpoints of the study were all-cause death, VTE recurrence, and bleeding events. VTE recurrence was defined as a new thrombus found by contrast-enhanced CT or ultrasound images of veins of the lower extremities. During the follow-up period, contrast-enhanced CT, and ultrasound images of veins of the lower extremities were performed when thrombosis recurrence was suspected, such as the appearance of shortness of breath or edema of the lower leg and/or an increase D dimer. In addition, contrast-enhanced CT was performed as a follow-up of the cancer according to the instructions of the oncologist. Acute hemorrhage, including at least one of fatal and symptomatic hemorrhage at important sites or organs (intracranial, intrathecal, intraocular, retroperitoneal, intra-articular, pericardial, and intramuscular hemorrhage accompanied by compartment syndrome); decrease in hemoglobin by ≥2 g/dL; and packed red blood cell transfusion of ≥2 units, are clinically apparent.^7)^

### Anticoagulant therapy

The anticoagulant therapy consisted of a self-administered subcutaneous injection or oral anticoagulant (warfarin and direct oral anticoagulant [DOAC]). The dose of heparin was adjusted so that the activated partial thromboplastin time was 1.5 times the control value, and the dose of warfarin was adjusted to maintain prothrombin time-international normalized ratio at 1.5–2.5. Rivaroxaban was administered twice per day at 15 mg for 21 days and then once per day at 15 mg. Apixaban was administered twice per day at 10 mg for 7 days and then twice per day at 5 mg. Edoxaban was administered once per day at 60 mg or at 30 mg for patients who weighed ≤60 kg; were also taking quinidine sulfate hydrate, verapamil hydrochloride, erythromycin, or cyclosporine; or had creatinine clearance of 30–50 mL/min. At the doctor’s discretion, some patients began treatment with rivaroxaban 15 mg once per day or apixaban 5 mg twice per day.

### Statistical analysis

Data normality was verified using the Kolmogorov–Smirnov test. Categorical data were expressed as absolute frequencies and percentages and were compared using the χ^2^ test. Continuous variables were expressed as means±standard deviation for normally distributed variables and as median values (25th–75th percentiles) for non-normally distributed variables and were compared using the Student’s t-test. The Kaplan–Meier method was used to compare the composite risk of all-cause death, major bleeding, and recurrent VTE between patients who were and were not receiving anticoagulant therapy. Statistical analyses were performed using the R statistical package version 3.1.0 (The R Foundation for Statistical Computing, Vienna, Austria; http://www.r-project.org/). A two-sided P value of <0.05 was considered statistically significant.

### Ethical statement

This study conformed to the ethical principles of the Declaration of Helsinki. The requirement for informed consent was waived because all data were anonymously cataloged. The institutional review board of the Musashino Red Cross Hospital approved the study’s protocol (the protocol number was 2058, and the date of approval by the ethics committee was October 1, 2020). The information disclosure document associated with this study is available on the hospital’s website. Patients were notified of their participation in the study and that they were free to opt out at any time.

## Results

**Figure 1:**
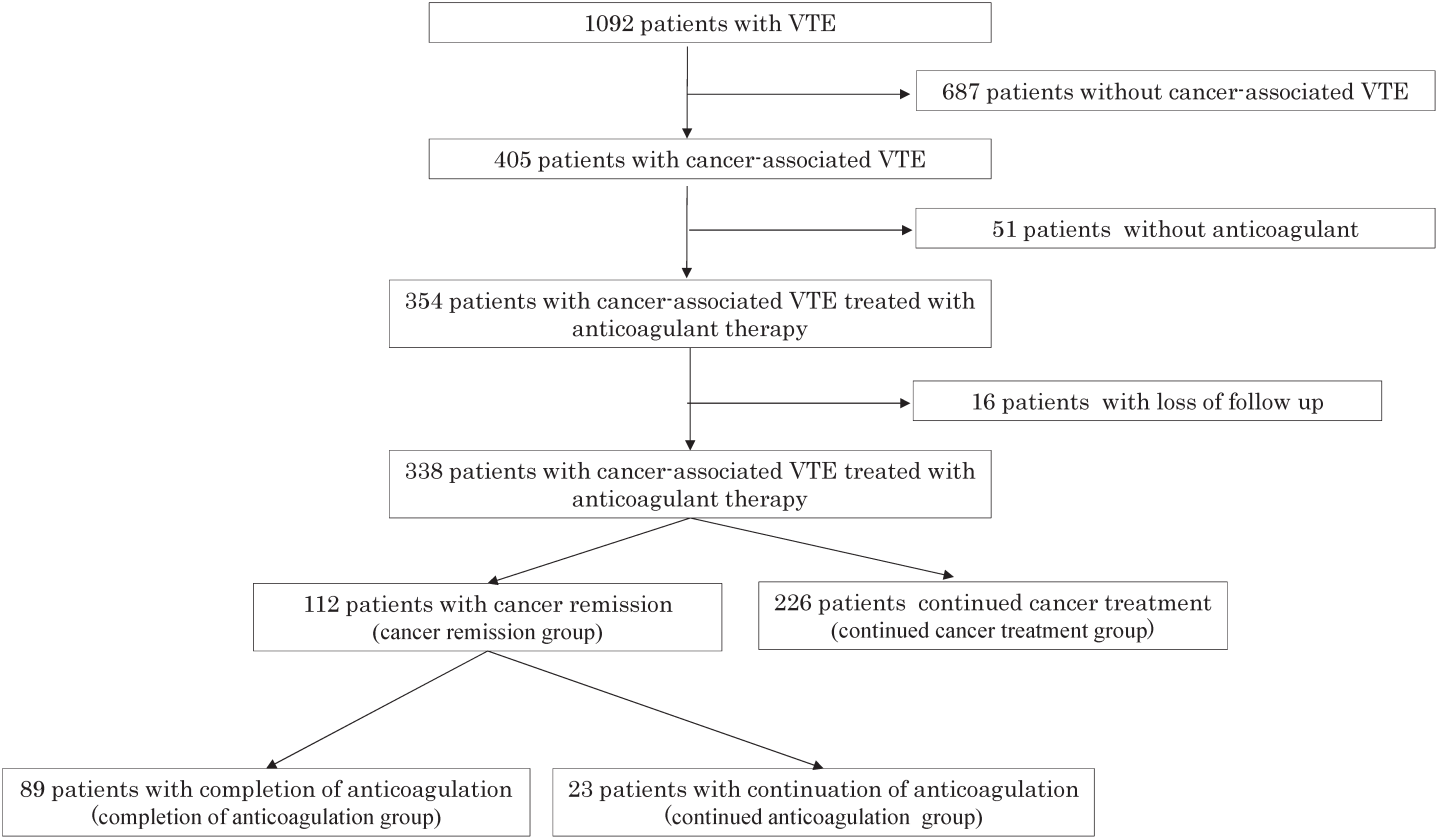
Fig. 1 Flowchart of participant inclusion.

From January 2012 to December 2019, 405 of 1,092 patients were diagnosed with cancer-associated VTE. Of these, 354 received anticoagulant therapy. After excluding 16 patients who could not be followed-up with, 338 patients were included in the analysis (**Fig. 1**).

VTE: venous thromboembolism

**Figure 2:**
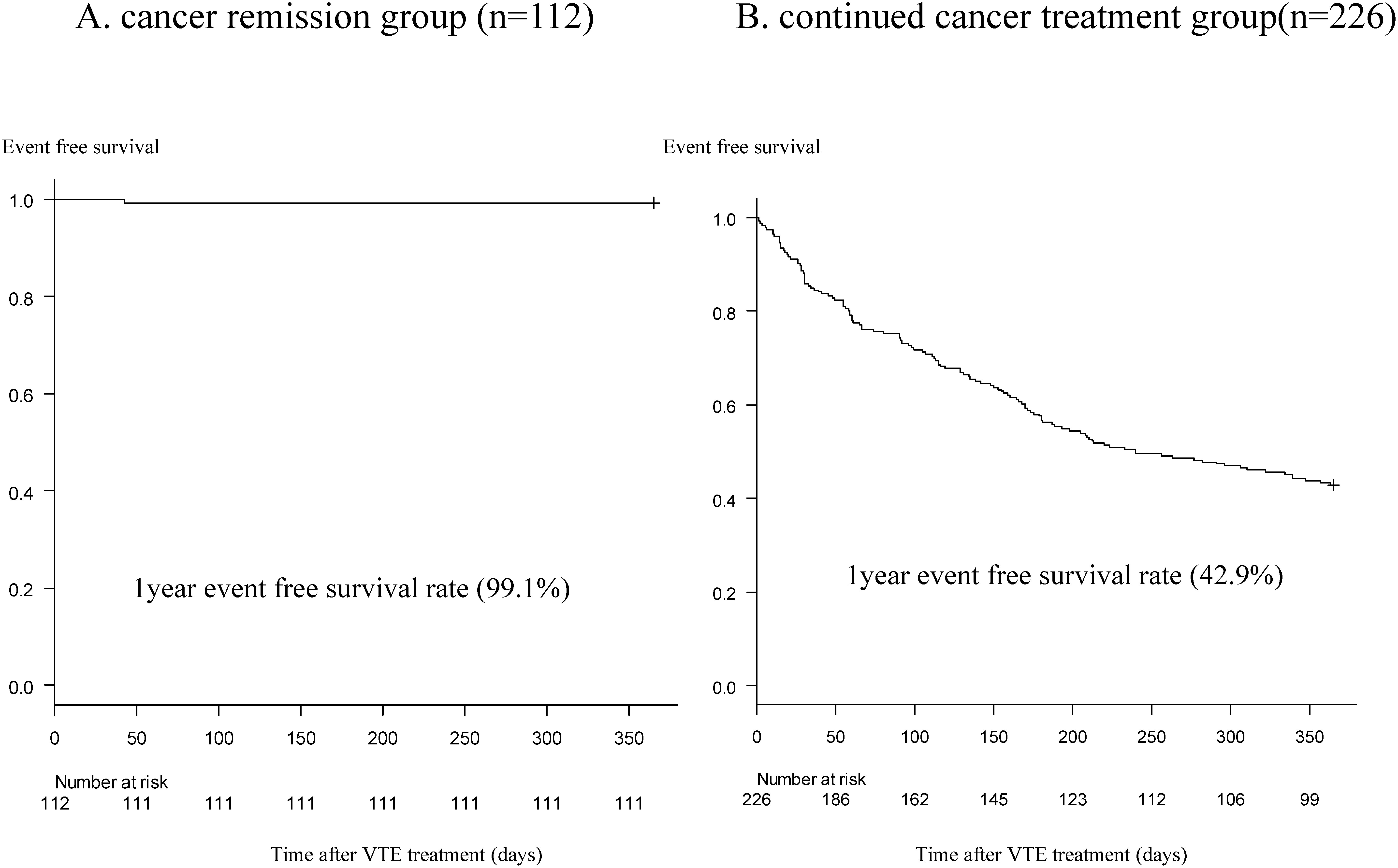
Fig. 2 Kaplan–Meier curve of estimated composite incidence of all-cause death and major bleeding and recurrent venous thromboembolism between the cancer remission and continued cancer treatment groups. The event-free survival rate was 0.9% in the cancer remission group and 42.9% in the continued cancer treatment group.

**Figure 2** shows the 1-year survival curve for cumulative incidence of all-cause death, VTE recurrence, and bleeding events with anticoagulant therapy. The event-free survival rate was 99.1% in the cancer remission group, with only one bleeding event. The rate in the continued cancer treatment group was 42.9%, with 79 deaths, 8 VTE recurrence cases, and 42 bleeding events. In addition, there were nine cases of fatal bleeding in this group.

**Figure 3:**
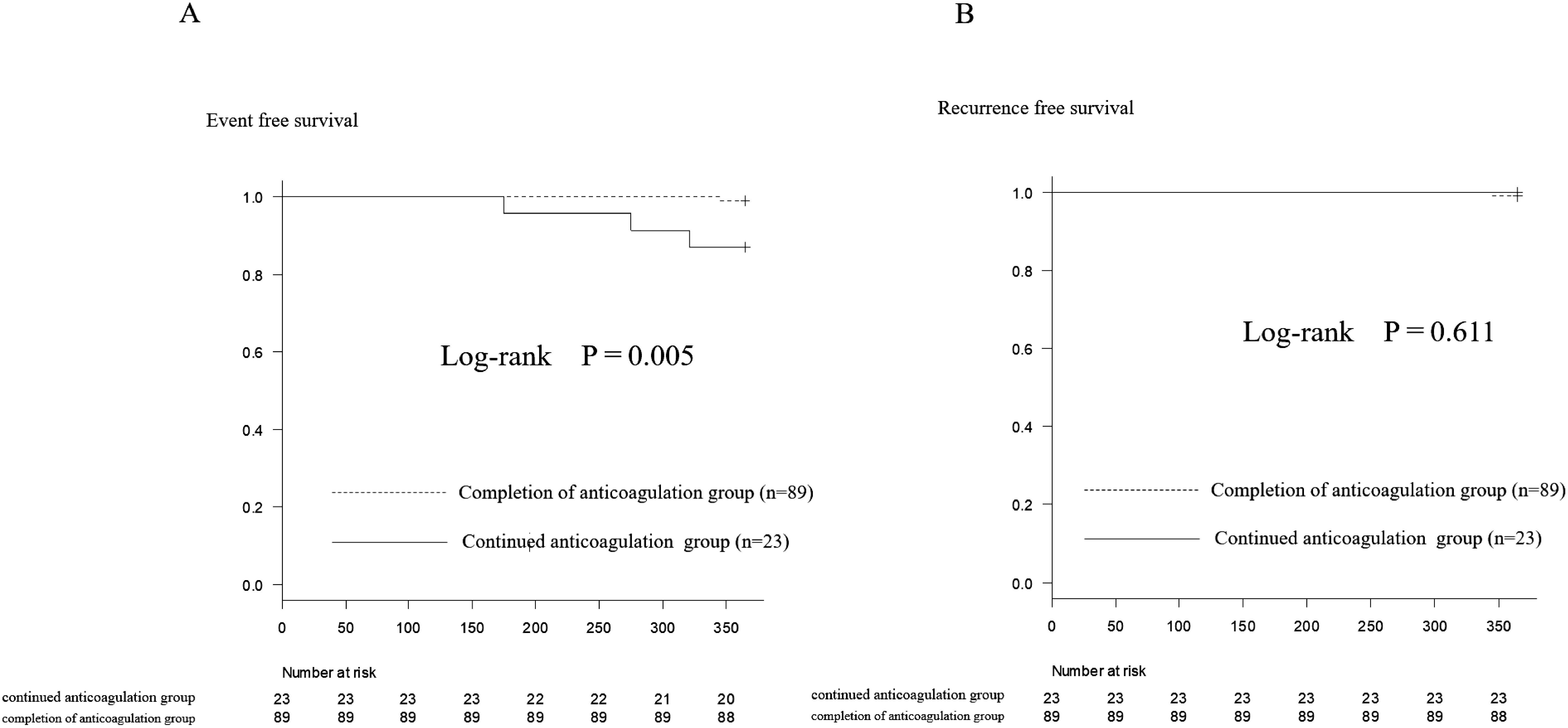
Fig. 3 Kaplan–Meier curve of estimated composite incidence of all-cause death and recurrent venous thromboembolism and bleeding between the completion of anticoagulation and continued anticoagulation groups. The event-free survival rate was 98.9% in the completion of anticoagulation group and 87% in the continued anticoagulation group. The completion of anticoagulation group had a lower incidence of composite events (**A**). No significant differences were observed for recurrent VTE between the two groups (log rank, P=0.611); the recurrence rate of the completion of anticoagulation group was 1.1% (**B**).

**Table 1** shows the demographic and clinical data of the patients in the cancer remission. The median duration of anticoagulation therapy in the completion of anticoagulation group (n=89) was 190 days. No significant differences were observed for age, sex, weight, comorbidities, laboratory data, or cancer stage. Warfarin was more commonly used in the continued anticoagulation group (n=23). The event-free survival rates were 98.9% and 87% in the completion of and continued anticoagulation groups, respectively (log rank, P=0.005). In the completion of anticoagulation group, there was one case of VTE recurrence and three cases of bleeding in the continued anticoagulation group (**Fig. 3A**). No significant differences were observed for VTE recurrence between two groups (log rank, P=0.611), but the recurrence rate of the completion of anticoagulation group was only 1.1% (**Fig. 3B**). There was no difference between warfarin and DOAC regarding all-cause death and recurrence after the end of anticoagulant therapy (log rank, 0.686).

**Table 1:** Table 1 Baseline characteristics of study participants in the cancer remission group

	Completion of anticoagulation group	Continued anticoagulation group	P value
Number	89	23	
Age (years)	68 [60.50, 77.50]	66 [53, 74]	0.233
Male (%)	70 (78.7)	17 (73.9)	0.588
Body weight (kg)	54 [47.80, 64]	58 [49, 67.50]	0.432
Comorbidities			
Hypertension (%)	36 (40.4)	14 (60.9)	0.101
Diabetes mellitus (%)	9 (10.1)	4 (17.4)	0.463
Dyslipidemia (%)	20 (22.5)	7 (30.4)	0.424
Heart disease (%)	5 (5.6)	2 (8.7)	0.631
Brain disease (%)	2 (2.2)	2 (8.7)	0.186
Smoking (%)	25 (28.1)	5 (21.7)	0.608
Antiplatelet therapy (%)	2 (2.2)	0 (0)	1
Pulmonary embolism (%)	26 (29.2)	9 (39.1)	0.45
Deep vein thrombosis (%)	80 (89.9)	21 (91.3)	1
Laboratory data			
White blood cell count (/µL)	6200 [600, 15700]	6800 [4000, 16200]	0.333
Hemoglobin (g/dL)	12.10 [5.70, 14.90]	11.70 [5.20, 15.60]	0.306
Platelet (×10^4^/µL)	24.20 [17.30, 29.80]	24.80 [18.65, 35.10]	0.443
D dimer (µg/mL)	2.80 [1.50, 6.35]	2.80 [1.75, 7.55]	0.876
Creatinine clearance (ml/min)	75 [61, 91]	74 [55, 86]	0.286
Anticoagulation			
Warfarin (%)	10 (11.2)	9 (39.1)	0.004
DOAC (%)	79 (88.8)	14 (60.9)	
Edoxaban	42	7	
Apixaban	28	5	
Rivaroxaban	9	2	
Duration of anticoagulant therapy (day)	190 [109, 365]	—	
Primary site of cancer			
Stomach	4	0	
Colorectum	17	6	
Esophagus	3	0	
Bile duct	1	0	
Gallbladder	2	1	
Lung	6	1	
Breast	4	2	
Uterus	23	7	
Ovary	20	2	
Prostate	3	1	
Kidney	0	3	
Blood	4	0	
Head	2	0	
Cancer stage (%)			
I	38 (45.8)	12 (52.2)	0.983
II	16 (19.3)	4 (17.4)	
III	22 (26.5)	6 (26.1)	
IV	7 (8.4)	1 (4.3)	
Treatment for cancer			
Surgery (%)	73 (82.0)	20 (87.0)	0.759
Chemotherapy (%)	36 (40.4)	9 (39.1)	1
Radiotherapy (%)	4 (4.5)	2 (8.7)	0.601

The data are expressed as numbers (%), means, and standard deviations, or medians and interquartile ranges.DOAC: direct oral anticoagulant

Anticoagulation in the continued cancer treatment group, 75.2% were on DOAC. Of the continued cancer treatment group, 33.2% completed their anticoagulation within the follow-up period. Bleeding was the reason for discontinuing anticoagulant therapy in 37.9% of patients. After the end of anticoagulant therapy, all-cause death was 23% and recurrences were 14.9%.

## Discussion

### Anticoagulation for cancer-associated VTE

In this study, all-cause death, VTE recurrence, and bleeding events were very common in patients with continued cancer treatment group. Whether or not the cancer was in remission was a very important point. Patients with VTE have been reported to have a high prevalence of cancer,^8)^ and VTE complications affect the survival prognosis.^9)^ Although anticoagulant therapy is the basic treatment for VTE, recurrence, and bleeding events are common in patients with cancer.^3)^ In Japan, health insurance does not cover low-molecular-weight heparin; thus, the anticoagulants used for patients with VTE with active cancer are either DOACs, warfarin, or unfractionated heparin. Recent studies have reported the usefulness of DOACs.^10–13)^ The American Society of Clinical Oncology^5)^ recommend DOAC over warfarin. In this study, there was also a lot of fatal bleeding in continued cancer treatment group. Bleeding events are reported to be more common in cancers of the bladder, stomach, and pancreas.^14)^ In anticoagulant therapy for cancer-associated VTE, monitoring for bleeding events is important, and clinicians face the dilemma of whether or not to use anticoagulant therapy.

### Anticoagulation after cancer remission

The American Society of Clinical Oncology^5)^ recommend that the duration of anticoagulant is at least 6 months. In the cancer remission group, there were no cases in which anticoagulant therapy was completed because of bleeding complications or drug side effects. JCS guidelines indicate that continuing anticoagulant therapy for 3 months is desirable even if the cause is transient and that long-term continuation is desirable for malignant tumors. Since chemotherapy also requires a certain period of treatment, it is necessary to take time to determine whether the cancer is in remission; therefore, the median duration of anticoagulant therapy was considered at 190 days.

Continuing anticoagulant therapy increases the risk of bleeding, whereas discontinuation increases the risk of VTE recurrence. The continued anticoagulation group may have been affected by the fact that more patients used warfarin, which is associated with more bleeding events than that in DOACs. In the continued anticoagulant therapy group, there were many cases before DOAC was approved for insurance, and it is thought that warfarin was used in many. This study found few cases of recurrence, even when anticoagulant therapy was discontinued after cancer remission. Japanese people experience more bleeding events than people from the West,^15)^ and cerebral hemorrhages are reported to be more common during anticoagulant therapy.^16)^ The same is true with antiplatelet therapy.^17)^ In other words, unnecessary antithrombotic therapy should be avoided.

VTE complications are less common in patients in whom cancer is in remission, and matching treatment with cancer therapy status is considered important.^18)^

However, because cancers that are in remission may recur, in addition to careful vigilance for VTE recurrence, cancer status needs to be observed. In addition, according to the JCS guidelines, recurrence rates vary depending on the severity of VTE and the site of the initial thrombus; reduced activities of daily living because of cancer therapy is also a risk factor for thrombus recurrence. Therefore, paying close attention to these factors is important.

### Limitation

First, this was a retrospective study conducted in a single institution. Therefore, selection bias was inevitable. A multicenter randomized study with a larger sample size is required to generalize our results. Second, CT or venous ultrasonography of the lower extremities is not used as follow-up in all cases. Third, the backgrounds of the patients were very diverse, and the number of patients in this study was too small to discuss the details, such as by cancer type.

## Conclusion

The outcomes of cancer-associated VTE greatly varied depending on whether the cancer was in remission. In addition, there were few recurrences when cancer was in remission, even if anticoagulant therapy was discontinued after a certain period.
